# HSC70 Inhibits Spring Viremia of Carp Virus Replication by Inducing MARCH8-Mediated Lysosomal Degradation of G Protein

**DOI:** 10.3389/fimmu.2021.724403

**Published:** 2021-09-29

**Authors:** Chen Li, Lin Shi, Yan Gao, Yuanan Lu, Jing Ye, Xueqin Liu

**Affiliations:** ^1^ State Key Laboratory of Agricultural Microbiology, College of Fisheries, Huazhong Agricultural University, Wuhan, China; ^2^ Hubei Engineering Technology Research Center for Aquatic Animal Diseases Control and Prevention, Wuhan, China; ^3^ Department of Public Health Sciences, University of Hawaii at Manoa, Honolulu, HI, United States; ^4^ State Key Laboratory of Agricultural Microbiology, College of Veterinary Medicine, Huazhong Agricultural University, Wuhan, China

**Keywords:** HSC70, SVCV G protein, MARCH8, complex, degradation, viral replication, ubiquitination

## Abstract

As a fierce pathogen, spring viremia of carp virus (SVCV) can cause high mortality in the common carp, and its glycoprotein (G protein) is a component of the viral structure on the surface of virion, which is crucial in viral life cycle. This report adopted tandem affinity purification (TAP), mass spectrometry analysis (LC-MS/MS), immunoprecipitation, and confocal microscopy assays to identify Heat shock cognate protein 70 (HSC70) as an interaction partner of SVCV G protein. It was found that HSC70 overexpression dramatically inhibited SVCV replication, whereas its loss of functions elicited opposing effects on SVCV replication. Mechanistic studies indicate that HSC70 induces lysosomal degradation of ubiquitinated-SVCV G protein. This study further demonstrates that Membrane-associated RING-CH 8 (MARCH8), an E3 ubiquitin ligase, is critical for SVCV G protein ubiquitylation and leads to its lysosomal degradation. Furthermore, the MARCH8 mediated ubiquitylation of SVCV G protein required the participation of HSC70 through forming a multicomponent complex. Taken together, these results demonstrate that HSC70 serves as a scaffold for MARCH8 and SVCV G, which leads to the ubiquitylation and degradation of SVCV G protein and thus inhibits viral replication. These findings have established a novel host defense mechanism against SVCV.

## Introduction

As a notifiable pathogen on the OIE-World Organization for Animal Health list, spring viremia of carp virus (SVCV) can cause spring viremia of carp (SVC), a hemorrhagic disease which is highly contagious in cyprinids (1), with the fatality rate up to 90% in spring at the water temperature within 10~17°C (2). However, no effective therapeutic treatment or anti-viral drug against SVCV infection has been found currently. SVCV, is a member of the genus *Spirivivirus* of *Rhabdoviridae family*, which is an enveloped, bullet-shaped virus. Its genome is composed of linear, negative-sense, and single-stranded RNA (ssRNA), with five genes encoding nucleoprotein, phosphoprotein, matrix protein, glycoprotein and RNA-dependent RNA polymerase, respectively, in the order of N–P–M–G–L (2, 3).

G protein of SVCV is the only protein on the surface of virion, which is the most important viral antigenic protein determining the infectious and serological properties of the virus (4). Previous studies have suggested that rhabdovirus G protein interacted with host factors to enhance viral endocytosis and membrane fusion (5). However, little is currently known about the host proteins which interact with SVCV G protein and how the interaction regulate SVCV replication.

Heat shock cognate protein 70 (HSC70), also known as HSPA8, is a member of the HSP70 protein family evolutionarily conserved with multiple functions, such as regulation of transcription, translation, and protein folding. HSC70 has been reported to participate in rotavirus and Japanese encephalitis virus entered by regulating the clathrin-mediated endocytosis (6, 7). Another important role of HSC70 is to regulate the replication of viral RNAs, such as hepatitis C virus (HCV) and influenza (8), which can also act as a molecular chaperone protein in the promotion for trafficking Coxsackievirus B3 (CVB3) (9). However, the role of HSC70 in SVCV infection remains unknown.

MARCH8, a member of RING-finger E3 ubiquitin ligases, belongs to the MARCH family, 11 cellular members of which have been identified in the mammal. The members of the MARCH family contain an RING-CH domain in N-terminal cytoplasmic tail potentially interacting with an E2 enzyme, which are similar to the other RING-type ubiquitin ligases. Generally, all of them have at least two transmembrane domains, except for MARCH7 and MARCH10 without any predicted transmembrane domains. In addition, C-terminal of which is of high variable. MARCH8 was originally identified as a cellular homolog of K3 and K5 of Kaposi’s sarcoma-associated herpesvirus, K3 of murine γ-2 herpesvirus 68, myxomavirus homolog M153R and other viral RING-CH E3 ligases (10, 11). It has been reported that MARCH8 can down-regulate the major histocompatibility complex class II (MHC-II) (12), the cluster of differentiation 44 (CD44) (13, 14), the cluster of differentiation 81(CD81) (13), the cluster of differentiation 86 (CD86) (15), the cluster of differentiation 98 (CD98) (14), Bap31 (13), IL-1 receptor accessory protein 5 and TRAIL receptor 1 (16). In addition to cellular proteins, MARCH proteins can target viral proteins. As a viral cofactor for enveloping Flaviviridae family members (17) particularly, two different antiviral mechanisms of MARCH8 have been reported: one is to trigger the viral glycoprotein degradation *via* lysosomes, and the other is to block its maturation in the Golgi (18, 19).

In this study, the host HSC70 protein was considered as a novel interactor of SVCV G protein. We deciphered that HSC70 negatively regulated SVCV replication *via* inducing lysosomal degradation of SVCV G protein. We further found that MARCH8 serves as an E3 ligase and forms a complex with SVCV G protein and HSC70. Knockdown of HSC70 significantly mitigated the interaction of MARCH8 with SVCV G protein, suggesting that as a scaffold protein, HSC70 interacts with both MARCH8 and SVCV G protein, allowing MARCH8-mediated ubiquitylation and SVCV G protein degradation to inhibit the replication of SVCV. These findings provide a new understanding of host defense mechanism against SVCV.

## Methods and Materials

### Cells and Virus

Fathead minnow (FHM) cells (ATCC^®^ CCL-42™) were kept in Medium 199 (M199, Hyclone, USA) at 28°C, supplemented with 10% fetal bovine serum (FBS, Gibco, Australia), for propagating SVCV (ATCC: VR-1390) in FHM cells at 28°C and harvested when over 80% virus-induced cytopathic effect (CPE) appeared. Following three cycles of freezing and thawing, culture media were collected from infected cells, centrifuged at 10,000g, 4°C, for 10 min to recover the supernatant, which was stored at -80°C.

### Plasmid Constructs

cDNA fragment encoding SVCV G protein (GenBank accession no.AJ318079.1) was amplified from the total RNA of SVCV-infected cells by RT-PCR, and then cloned into pcDNA4 or pEGFP-N1 vectors. Truncated mutant SVCV G protein lacking CT (pcDNA4-G-ΔCT) was generated by introducing deletions of amino acids 488-509. The SVCV G protein mutants K to R were constructed by changing the indicated amino acid(s) to arginine (R) by with overlap extension PCR. DNA fragment encoding HSC70-Flag was subcloned into pcDNA4 vector to construct pcDNA4-HSC70-Flag plasmid. The coding sequence of zebrafish MARCH8 was cloned into pcDNA4. The MARCH8 mutants W to A were constructed by changing the indicated amino acid(s) to alanine (A) by with overlap extension PCR. To generate the RFP-HSC70, RFP-RAB5, RFP-RAB7 and RFP-MARCH8 expression plasmids, the cDNA fragments encoding zebrafish HSC70, zebrafish RAB5, zebrafish RAB7 and zebrafish MARCH8 were cloned into the pDsRed1-C1 vector. Ubiquitin plasmids HA-ub, HA-ub-K48 and HA-ub-K63 were obtained from Professor Jianguo Su (College of Fisheries, Huazhong Agricultural University). All the constructs have been confirmed by DNA sequencing. Primers including endonuclease cleavage sites for plasmid construction are shown in **Supplementary Table 1**.

### Reagents and Antibodies

MG132, 3-MA, NH_4_Cl, bafilomycin A1 (Baf-A1) and chloroquine (CQ) from Sigma-Aldrich were used at a final concentration of 10 μM, 10 mM, 15 mM, 50nM and 50 μM, respectively, as well as CHX and actinomycin D from MCE at a final concentration of 10 μg/mL, with mouse monoclonal anti-SVCV G protein generated in our lab. Commercially available antibodies used included mouse monoclonal antibodies against Flag and His, rabbit polyclonal antibodies against ACTB and HSC70, horseradish peroxidase-conjugated anti-mouse and anti-rabbit IgGs (Abclonal Technology), rabbit anti-polyubiquitin mAb, rabbit anti-K48-polyubiquitin mAb and rabbit anti-K48-polyubiquitin mAb (Epitomics).

### qRT-PCR

Total RNA was extracted with TRIzol Reagent (TAKARA) by the manufacturer’s instruction. The reverse transcription was conducted with the ReverTra Ace qPCR RT kit (TAKARA), with relative cDNA expressions determined by qRT-PCR with TB Green Realtime PCR Master Mix (TAKARA). Amplification was performed at 95°C for 5 min, followed by 40 cycles of that at 95°C for 15 s, at 60°C for 20 s, and at 72°C for 20 s. Fluorescent signals were studied through a Light Cycler/Light Cycler 480 System (Roche), with relative mRNA levels calculated by 2-^ΔΔCT^ method (with CT as threshold cycle). Primers employed in this study are listed in **Table 1**.

**Table 1:** Primers of this study.

Application	Prime Name	Sequence (5′–3′)
qRT-PCR	qTBP-F	TTACCCACCAGCAGTTTAG
qRT-PCR qRT-PCR qRT-PCR qRT-PCR qRT-PCR	qTBP-R qCc40S-F qCc40S-R qMARCH8-F qMARCH8-R	ACCTTGGCACCTGTGAGTA CCGTGGGTGACATCGTTACA TCAGGACATTGAACCTCACTGTCT CTCGGTCACTTTCCACG CTTGAGTCTCCTCCACAACT
qRT-PCR	SVCV-G-F	CGACCTGGATTAGACTTG
qRT-PCR	SVCV-G-R	AATGTTCCGTTTCTCACT
qRT-PCR	SVCV-M-F	TACTCCTCCCACTTACGA
qRT-PCR qRT-PCR qRT-PCR qRT-PCR qRT-PCR qRT-PCR qRT-PCR qRT-PCR qRT-PCR Plasmid construction Plasmid construction Plasmid construction Plasmid construction Plasmid construction Plasmid construction Plasmid construction Plasmid construction Plasmid construction Plasmid construction Plasmid construction Plasmid construction Plasmid mutation Plasmid mutation Plasmid mutation	SVCV-M-R SVCV-N-F SVCV-N-R qISG15-F qISG15-R qPKR-F qPKR-R qViperin-F qViperin -R HSC70-Flag-F HSC70-Flag-R HSC70-His-F HSC70-His-R MARCH8-His-F MARCH8-His-R MARCH8-Flag -F MARCH8-Flag-R SVCV G-F SVCV G-R SVCV G-EGFP-F SVCV G-EGFP-R SVCV G-△CT-F SVCV G-△CT-R SVCV G-KR-F	CAAGAGTCCGAGAAGGTC GCGGTTTTCTGTATGTGTCTC CTCTGCCAAATCACCATACTC TAATGCCACAGTCGGTGAA AGGTCCAGTGTTAGTGATGAGC ACCTGAAGCCTCCAAACATA GCATTCGCTCATCATTGTC GCAAAGCGAGGGTTACGAC CTGCCATTACTAACGATGCTGAC CCCAAGCTTGCCACCATGTCCAAGGGACCAGCTGTTGGTATT CCGCTCGAGTTACTTATCGTCGTCATCCTTGTAATC GTCGACCTCCTCGATGGTTGGG CCCAAGCTTGCCACCATGTCCAAGGGACCAGCTGTTGGTATT CCGCTCGAGGTCGACCTCCTCGATGGTTGGG CGGAATTCATGAACATGCCACTGCACCAGATCT CCGCTCGAGCACGTGAAGGATCTCCATACTG CGGAATTCATGAACATGCCACTGCACCAGATCT CCGCTCGAGTCACTTATCGTCGTCATCCTTGTAATC CACGTGAAGGATCTCCATACTG CCCAAGCTTGCCACCATGTCTATCATCAGCTACATCGCAT CCGCTCGAGAACTAAAGACCGCATTTCGTGT CCGCTCGAGGCCACCATGTCTATCATCAGCTACATCGCAT CCCAAGCTTAACTAAAGACCGCATTTCGTGT CCCAAGCTTGCCACCATGTCTATCATCAGCTACATCGCAT CCGCTCGAGAGCAACACAGCATCTGATGAGAAGA CCCAAGCTTGCCACCATGTCTATCATCAGCTACATCGCAT
Plasmid mutation Plasmid mutation Plasmid mutation Plasmid mutation Plasmid mutation Plasmid construction Plasmid construction Plasmid construction Plasmid construction	SVCV G-KR-R SVCV G-K493R-F SVCV G-K493R-R SVCV G-K496R-F SVCV G-K496R-R SHVV-G-F SHVV-G-R IHNV-G-F IHNV-G-R	CCGCTCGAGAACTAAAGACCGCATTTCGTGTGATTCTGTTGCAGGCCGTCTACTCCTCCTCATCAAATA CCCAAGCTTGCCACCATGTCTATCATCAGCTACATCGCAT CCGCTCGAGAACTAAAGACCGCATTTCGTGTGATTCTGTTGCAGGC CGTTTACTCCTCCTCATCAAATA CCCAAGCTTGCCACCATGTCTATCATCAGCTACATCGCAT CCGCTCGAGAACTAAAGACCGCATTTCGTGTGATTCTGTTGCAGGCCGTCTACTCCT CGGAATTCGCCACCATGAAATCAATCATTGCACTTACGT GCTCTAGAGGGAACAAATTGATACTGCTGCAAA CCCAAGCTTGCCACCATGTACACCATGATCACCACTCCGC CCGCTCGAGGGACCGGTTTGCCAGGTGATACATG

### Plasmid Transfection and Virus Infection

Recombinant plasmids were transfected with FuGENE HD (Promega) following the protocol provided by the manufacturer. For virus infection assays, cells were infected with SVCV (0.05 MOI) at 28°C. After virus absorption for 1 h,PBS was adopted to wash the cells for three times, which were subsequently maintained in M199 with 5% FBS.

### Co-Immunoprecipitation and Western Blot Analyses

Co-immunoprecipitation (Co-IP) assay was conducted with Pierce Co-IP Kit (Thermo Fisher Scientific) by the manufacturer’s instruction. Through the immunoblot analysis, cells were obtained with ice-cold PBS, and lysed with IP lysis/washing buffer on ice for 5 min. After 10-minute centrifugation at 13,000g, the supernatant was collected, to separate immunoprecipitants or whole-cell extracts with 10% SDS-PAGE, which were transferred onto a polyvinylidene difluoride membrane (Bio-Rad). Cell membranes were sealed with 2% bovine serum albumin (BSA) at room temperature for 1 h, and subsequently incubated with mouse anti-His (ABclonal, China, 1:3000 dilution), mouse anti-Flag (ABclonal, China, 1:3000 dilution), rabbit anti-β-actin (ABclonal, China, 1:10000 dilution), or anti-SVCV-G monoclonal antibodies for 2 h, with rabbit anti-β-actin antibodies as internal controls. The membranes were washed with TBST, and then incubated for 45 min with horse radish peroxidase-conjugated secondary goat anti-mouse or anti-rabbit (ABclonal, China, 1:2000 dilution) antibodies. Finally, reactive proteins were detected by chemical luminescence substrate (General Electric, USA) with Amersham Imager 600 (GE, USA).

### Confocal Laser Scanning Microscopy Assays

FHM cells were sowed in a 20-mm dish, and then transfected with indicated plasmids. Following the incubation at 28°C for 24 h, the cells were fixed with 4% PFA for 1 h after washing with PBS twice, with the cell nuclei stained by 4,6-diamidino-2-phenylindole (DAPI), which were washed for three times. Images were obtained with a confocal microscope (Leica; Germany).

### RNA Interference

To knock down HSC70 expression, the three pairs of either HSC70-specific siRNAs (GenePharma, China) were transfected with RNA interference assay. FHM cells were sowed in a 12-well plate, and then transfected with 100 nM siRNA against HSC70 (siHSC70) or negative control (NC) with Lipofectamine 2000 at 24 h post infection (hpi) with SVCV (MOI = 0.05). At another 24 hpi, total RNA was isolated and processed qRT-PCR. HSC70-specific siRNA sequences were as follows:

siRNA1: Forward: 5’- CCAAGGAUGCUGGAACCAUTT-3’; Reverse: 5’-AUGGUUCCAGCAUCCUUGGTT-3’; siRNA2: Forward: 5’- GCUCCAAGACUACUUCAAUTT-3’; Reverse: 5’-AUUGAAGUAGUCUUGGAGCTT-3’; siRNA3: Forward: 5’- GCAGAAGGAGCUGGAGAAATT-3’; Reverse: 5’-UUUCUCCAGCUCCUUCUGCTT-3’. NC siRNA sequences were as follow: NC: Forward: 5’-UUCUCCGAACGUGUCACGUTT-3’; Reverse: 5’-ACGUGACACGUUCGGAGAATT -3’.

### Plaque Assay

Virus titer was determined by plaque assay with FHM cells, which were briefly sowed in 12-well plates and allowed to become confluent. A 10-fold diluted SVCV stock was sowed onto cell monolayers. After adsorption at 28°C for 1 h, serum-free M199 was adopted to wash the cells, which were covered with an overlay medium with 3% FBS and 1.5% carboxymethyl cellulose (CMC, Sigma-Aldrich). After infection for 72 h, infected cells were fixed with 10% paraformaldehyde and stained with 0.5% crystal violet. After washing vigorously with running water, visible plaques and viral titers were calculated. All data were presented as the mean of triplicated samples.

### Statistics Analysis

All experimental tests in this study were repeated three times or more. Data were represented as mean ± SD (standard deviation). The difference between two groups was compared through the two-way ANOVA test. GraphPad Prism 7.0 was adopted for statistical analyses and calculations. p value indicates significant differences. * represents p<0.05 while ** represents p<0.01.

## Results

### SVCV G Protein Interacts With the Host Protein HSC70

In our previous study, host proteins potentially interacting with SVCV G protein were screened through the TAP-MS system (20). HSC70 (Gene ID: NM_001110403.1) involved in the replication of several viruses was selected for further study (21, 22).

**Figure 1:**
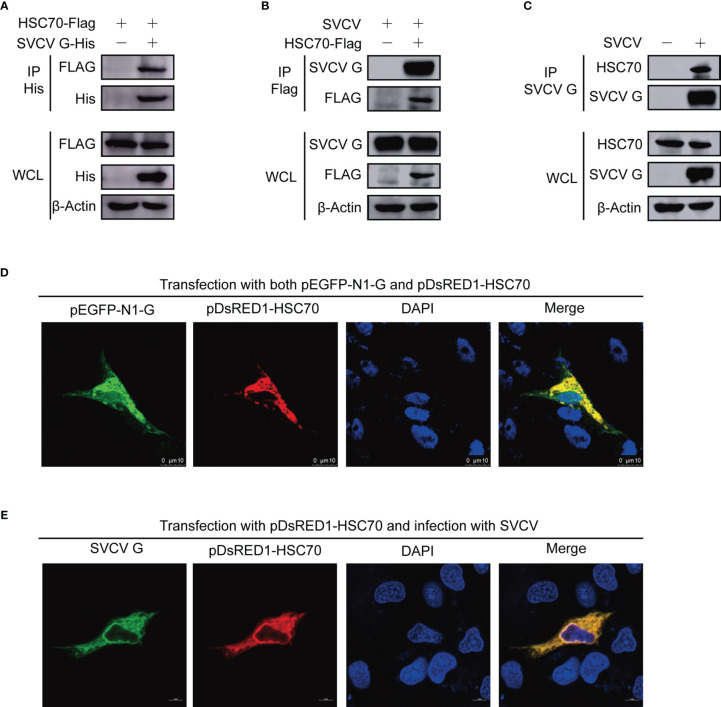
Interaction between SVCV G and HSC70 proteins. **(A)** Interaction between SVCV G and exogenous HSC70 proteins. FHM cells were transfected with expressed plasmids. After transfection for 24 h, prepared cell lysates were immunoprecipitated (IP) by anti-His antibodies and immunoblotted by anti-Flag or anti-His antibodies. **(B)** Interaction between SVCV G and exogenous HSC70 proteins in SVCV-infected cells. FHM cells were transfected with expressed plasmids. After transfection for 24 h, the MOI of SVCV-infected cells was 0.05. At 36 hpi, prepared cell lysates were immunoprecipitated by anti-FLAG antibodies and immunoblotted by anti-G or anti-FLAG antibodies. **(C)** Interaction between SVCV G and endogenous HSC70 protein in SVCV-infected cells. The MOI of SVCV-infected FHM cells was 0.05. At 36 hpi, prepared cell lysates were immunoprecipitated by anti-G antibodies and immunoblotted by anti-G or anti-HSC70 antibodies. **(D)** Localization of SVCV G and exogenous HSC70 proteins in FHM cells. FHM cells were sowed onto 6-well plates, and cultured with coverlips in the wells overnight, which were then transfected with pDsRed-HSC70 and pEGFP-G. After transfection for 24 h, 4% paraformaldehyde was added to fix the cells. After washing, the nuclei were backstained with DAPI (blue), to obtain the samples for the observation with a confocal laser scanning microscope. **(E)** Colocalization of SVCV G with exogenous HSC70 in SVCV-infected cells. FHM cells were transfected with the indicated expression plasmids. After transfection for 24 h, cells were mock infected or infected with SVCV at an MOI of 0.05. At 24 hpi, cells were fixed and probed with mouse anti-SVCV G antibody. Nuclei were counterstained with DAPI (blue). The cell samples were observed with a confocal laser scanning microscope.

To further validate the interaction of HSC70-SVCV G protein, FHM cells were transfected with pcDNA4-G and/or pcDNA4-HSC70-Flag through the immunoprecipitation assay. Therefore, pcDNA4-HSC70-Flag was detected in the precipitate when FHM cell lysates expressing both pcDNA4-HSC70-Flag and pcDNA4-G were immunoprecipitated with His antibody, indicating that SVCV G protein can interact with exogenous HSC70 in FHM cells (**Figure 1A**). For further substantiating HSC70 binding to SVCV G protein, FHM cells were transfected with pcDNA4-HSC70-Flag, which were then infected with SVCV through a immunoprecipitation assay performed with anti-FLAG antibodies. The findings showed that SVCV G protein was easily detected in the precipitate, indicating that HSC70 can interact with SVCV G protein (**Figure 1B**). To further determine the interaction of SVCV G protein with HSC70 under physiological conditions, FHM cells were infected through an immunoprecipitation assay with anti-G mono-antibodies. Thus, HSC70 was detected in lysates of SVCV-infected cells, but not in simulated infection control cells (**Figure 1C**). These results clearly showed that SVCV G protein interacted with HSC70 of host cells.

To detect the subcellular localization of HSC70 and SVCV G protein, confocal microscopy assays were performed with FHM cells, which were transfected with pDsRed1-C1-HSC70 and pEGFP-N1-G, thereby detecting HSC70 and SVCV G protein to be colocalized in the cytoplasm (**Figure 1D**). To confirm the interaction, the colocalization of HSC70 with SVCV G protein was further examined in SVCV-infected FHM cells. We transfected FHM cells with pDsRed1-C1-HSC70, following infection with SVCV at a multiplicity of infection (MOI) of 0.05, and an immunofluorescent-antibody assay (IFA) was performed by using anti-G mono-antibody. As shown in the result, HSC70 was also colocalized with SVCV G protein in the cytoplasm of SVCV-infected cells (**Figure 1E**). These results strongly support that SVCV G protein interacts with HSC70 in cells.

### HSC70 Negatively Modulates SVCV Multiplication

**Figure 2:**
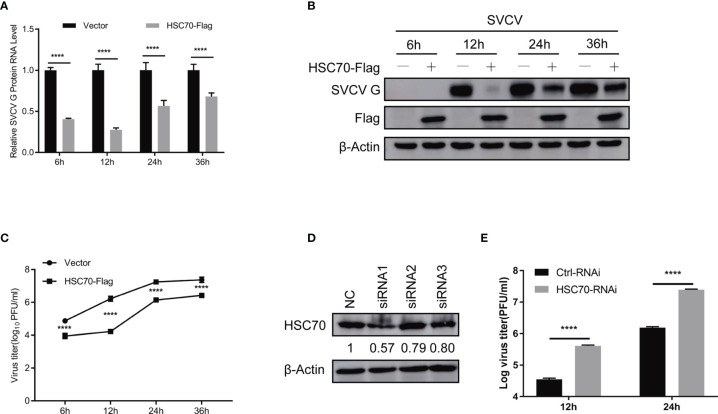
Negative modulation of HSC70 on SVCV replication. **(A–C)** Inhibition of HSC70 overexpression on SVCV growth in FHM cells. FHM cells were transfected with HSC70 plasmid and empty vector (2μg each) for 24 h, with an MOI of 0.05 after SVCV infection. At the indicated time points, cell samples and the supernatant were collected for the analysis of qRT-PCR **(A)**, Western blot **(B)**, or plaque formation assay **(C)**. **(D)** RNA interference effect on the expression of endogenous HSC70. FHM cells were transfected with HSC70-specific siRNAs (siRNA1, siRNA2, and siRNA3) or control siRNA (NC) for 24 (h) The efficiency of siRNAs to inhibit the HSC70 expression was detected by Immunoblot. siRNA1 showed the best silencing efficiency. Relative band quantification (below Western blot) was determined by ImageJ, by normalizing to loading control, β-actin. **(E)** Effects of HSC70 silencing on SVCV replication. FHM cells were dealt with HSC70 or control RNAis, with an MOI of 0.05 after SVCV infection. Viral titers in the supernatant were determined by plaque assay at different time points (12 and 24 h) after SVCV infection. All data represented three separate experiments. ****p < 0.0001.

To evaluate the role of HSC70 in response to SVCV infection, both qRT-PCR and immunoblot assays were performed. Ectopic expression of HSC70 significantly resulted in decreased expression of SVCV RNA level (**Figure 2A**) and G protein level (**Figure 2B**) at different infection time-points, respectively. Consistently, the production of infectious SVCV in the supernatants of HSC70-overexpressed cells was significantly reduced at different post infection times, as assessed by plaque formation assay (**Figure 2C**).

To consolidate our above findings, siRNA against HSC70 (siHSC70) or negative control siRNAs (NC) was adopted to transfect into FHM cells. The silencing efficiency of 3 siRNAs for HSC70 was examined, and the siRNA1 showed best knockdown efficiency was selected for the following experiments (**Figure 2D**). Subsequently, FHM cells were transfected with siHSC70 or NC followed by infection with SVCV. When compared to negative control cells, knockdown of HSC70 caused marked increase of the SVCV production (**Figure 2E**). Taken together, these data indicate a critical role of HSC70 in modulating the viral infection.

### HSC70 Does Not Affect the Attachment, Entry and Genomic Transcription of SVCV

**Figure 3:**
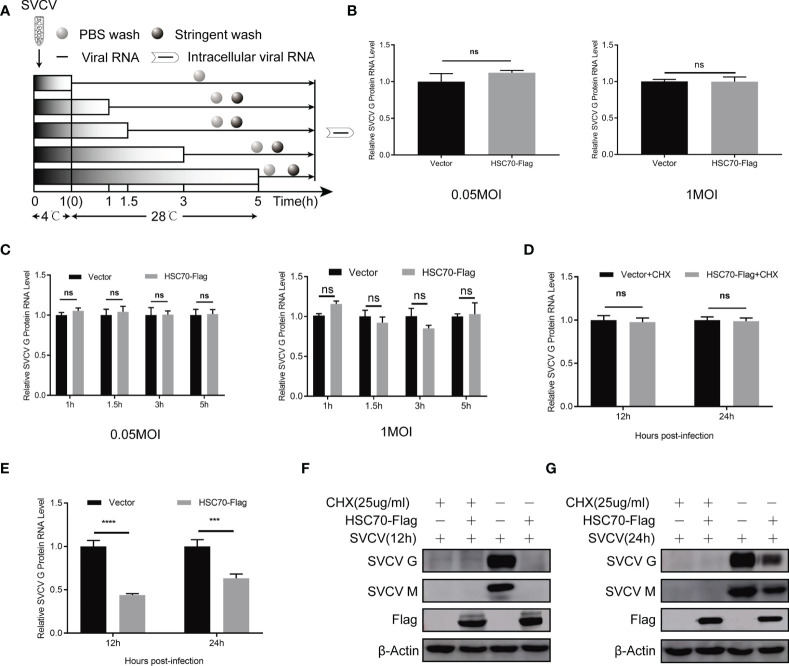
No effect of HSC70 in the early stages of SVCV entry and transcription. **(A)** Overview of experimental design for examination of virus attachment and entry. **(B)** Effect of HSC70 on SVCV attachment by qRT-PCR in FHM cells. **(C)** Effect of HSC70 on SVCV entry by qRT-PCR in FHM cells. Intracellular viral RNA was quantified from intracellular viral RNAs at given time points by qRT-PCR. **(D, E)** Effect of HSC70 on SVCV transcription. After transfection for 24 h, indicated plasmids-transfected FHM cells were infected with SVCV, with an MOI of 0.05, and in the absence or presence of CHX (25ug/ml) for 12h or 24h. The accumulation of SVCV G mRNA transcripts were analyzed by qRT-PCR. All data represented three separated experiments. ***p < 0.001, ****p < 0.0001. ns, no significant differences. **(F, G)** Immunoblot analysis of G and M proteins described in **(D, E)**.

To explore at which step of viral life cycle the HSC70 affects SVCV infection, we firstly examined the effect of HSC70 on the attachment of G cells and viral entry (**Figure 3A**). To analyze the function of HSC70 in SVCV attachment, FHM cells overexpressing HSC70 were incubated with the same amount of SVCV (MOI of 0.05 and MOI of 1) at 4°C, so that the virus could attach without entering the cell surface. After 1 h of incubation, PBS was adopted to wash the cells for removing unattached virus, and the number of virion attached to the cell surface was quantified by qRT-PCR. The findings showed that no significant difference viral attachment occurred in FHM cells transfected with HSC70 plasmids compared with cells transfected by pcDNA 4.0 plasmids (empty vector) (**Figure 3B**). To explore the impact of HSC70 on SVCV entry, FHM cells were incubated at 28°C for initial viral entry. Infected cells were stringently washed with an alkaline high-salt solution at different time points (1, 1.5, 3, 5h), to separate free virus and cell surface-associated virus. Intracellular viral RNAs were quantified by qRT-PCR. In agreement with the results of attachment, viral RNA levels in cells transfected by HSC70 plasmids showed no significant difference with those detected from control cells transfected with empty vector at 1 to 5 h post-infection (**Figure 3C**). These results suggest that HSC70 does not affect the attachment and entry step of SVCV.

HSC70 inhibition of SVCV infection may be a direct destruction of the virus transcription. Therefore, we used qRT-PCR to analyze the accumulation of SVCV mRNA. As shown in **Figures 3D, E**, overexpression of HSC70 in SVCV-infected FHM cells did not affect the level of viral mRNA after treatment with CHX drugs. At the same time, in order to ensure the effectiveness of CHX processing, Immunoblots of cell lysates were probed by mouse mAbs to G and M respectively (**Figures 3F, G**). This indicates that HSC70 did not directly affect the virus transcription or the stability of the viral mRNA.

### HSC70 Affect the Stability of Viral Protein by the Lysosomal Pathway

**Figure 4:**
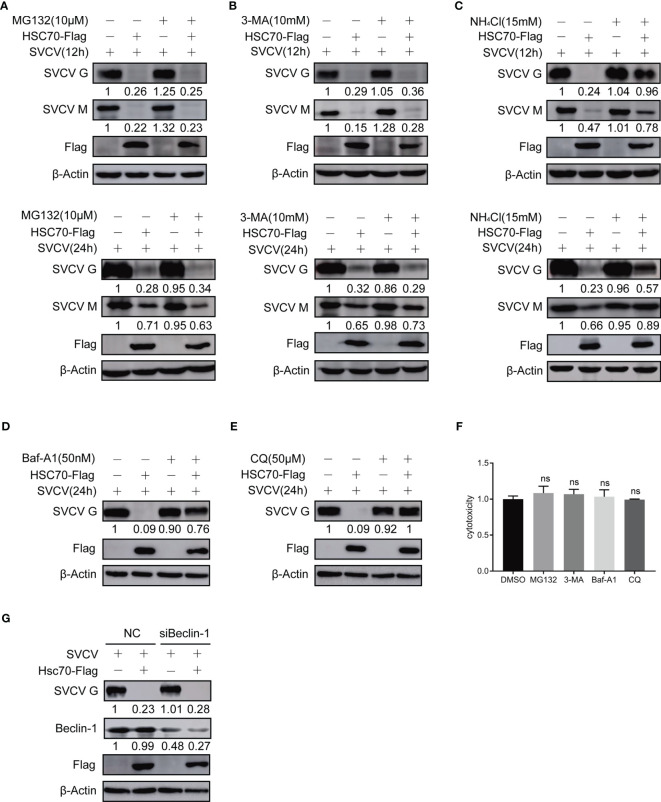
Effect of HSC70 on viral protein stability *via* lysosomal pathway. **(A–E)** After transfection for 24 h, indicated plasmids-transfected FHM cells were infected with SVCV, with an MOI of 0.05. At 6 or 18 hpi, the cells were treated with 10μM MG132 **(A)**, 20mM 3-MA **(B)**, 15 mM NH4Cl **(C)**, 50 nM bafilomycin A1 (Baf-A1) **(D)**, 50 μM chloroquine (CQ) **(E)** or DMSO for 6 h, and protein levels were examined by Western blot with corresponding antibodies. **(F)** Cells were incubated with inhibitor for 6 hours before assessment of cytotoxicity (fluorescence). **(G)** Knockdown of Beclin-1 cannot mitigating HSC70-induced degradation of SVCV G protein. Cells were treated by Beclin-1 or control siRNA with pcDNA4-HSC70 or pcDNA4. After transfection for 6 h, cells were infected by SVCV, with an MOI of 0.05. At 24 hpi, prepared cell lysates were immunoblotted by anti-Beclin-1, anti-Flag or anti-G antibodies. ns, no significant differences.

Previous studies have shown that HSC70 can shorten the half-life or reduce the stability of its client proteins (23). To assess whether the cellular protein HSC70 affects viral proteins’ synthesis or stability during the infection of SVCV, proteasome inhibitor MG132, lysosome inhibitor NH4Cl, bafilomycinA1 (Baf-A1) and chloroquine (CQ), and autophagosome inhibitor 3-MA were exployed for treating SVCV-infected FHM cells in HSC70, respectively. The cytotoxicity of the inhibitors against FHM cells was determined using the MTT assay. All doses of the inhibitors used in the experiments showed no detectable cytotoxicity (**Figure 4F**). The results revealed that the concentrations of viral G and M proteins in HSC70-tranfected cells were lower than those in untreated cells. In the presence of HSC70, further treatment with MG-132 not only failed to restore, but also further significantly reduced the relative levels of viral G and M proteins (**Figure 4A**). The changes of viral proteins in 3-MA treated cells were similar to those in MG132-treated cells (**Figure 4B**). Furthermore, the expressions of viral proteins, which were reduced due to HSC70 overexpression, restored in SVCV-infected cells in the presence NH4Cl, bafilomycin A1 (Baf-A1) and chloroquine (CQ) (**Figures 4C–E**). To exclude the effect of autophagy on viral protein reduction, FHM cells were transfected with Beclin-1-specific siRNA to interfere with autophagy. In Beclin-1 knockdown cells, the reduction of viral protein mediated by HSC70 cannot be achieved to restore (**Figure 4G**).These findings suggest that chaperone protein HSC70 reduced the stability of SVCV-infected viral proteins by promoting lysosomal clearance of SVCV.

### HSC70 Induces SVCV G Protein Degradation in a Lysosome-Dependent Manner

**Figure 5:**
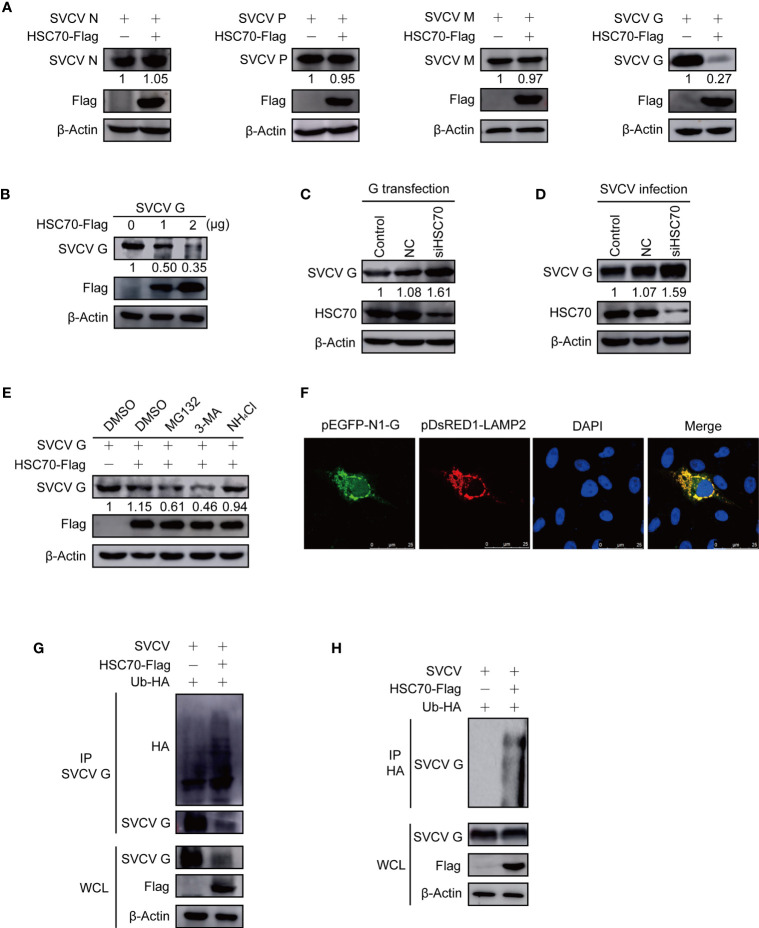
Induction of HSC70 on SVCV G protein degradation in a lysosome-dependent manner. **(A)** The indicated plasmids were adopted to transfect FHM cells. The expressions of pcDNA4-HSC70-Flag and viral proteins were determined by Western blot. **(B)** Degrade of HSC70 overexpression on SVCV G protein in a dose-dependent manner. The number of pcDNA4-HSC7-Flag plasmid was increased by transfecting FHM cells with pcDNA4-G plasmid. After transfection for 24 h, protein levels were examined by Western blot. **(C)** FHM cells were dealt with HSC70 or control RNAis, and transfected with pcDNA4-G. After 24 h of transfection, cell lysates were prepared, and the expressions of SVCV G protein were examined by Western blot. **(D)** FHM cells treated with HSC70 or control RNAis were infected with SVCV, with an MOI of 0.05. At 24 hpi, cell lysates were prepared, and the expression of SVCV G protein was examined by Western blot. **(E)** Effects of inhibitors on HSC70-mediated degradation of SVCV G protein. FHM cells sowed in 6-well plates overnight were co-transfected with indicated plasmids. After 18 h of transfection, the cells were dealt with indicated inhibitors for 6 h, followed by the immunoblot analysis. **(F)** Localization of SVCV G protein to lysosome. FHM cells were briefly co-transfected with lysosomal protein markers pEGFP-N1-G and LAMP2-RFP, respectively, and sowed on observation plates for confocal microscopy. After 24 h, the cells were stained with DAPI after fixing with 4% (v/v) paraformaldehyde. All samples were then examined under a confocal microscope. Green represents SVCV G protein overexpression and red stands for LAMP2 overexpression. Blue staining indicates nucleus. Yellow indicates the co-localization between SVCV G protein and organelle in the merged images. **(G)** Overexpression of HSC70 enhances the ubiquitylation of SVCV G protein. Plasmids encoding HA-ubiquitin and pcDNA4-HSC70-Flag or pcDNA4 were used to transfect FHM cells as controls. After transfection for 6 h, the cells were infected by SVCV, with an MOI of 0.05. At 18 hpi, the cells were dealt with 15mM NH4Cl for 6 (h) Prepared cell lysates were immunoprecipitated by anti-G antibodies and immunoblotted by anti-G or anti-HA antibodies. All the cell lysates were examined by Western blot for protein expressions (bottom panels). **(H)** Overexpression of HSC70 enhances the ubiquitylation of SVCV G protein. Plasmids encoding HA-ubiquitin and pcDNA4-HSC70-Flag or pcDNA4 were used to transfect into FHM cells as controls. After transfection for 6 h, the cells were infected by SVCV, with an MOI of 0.05. At 18 hpi, the cells were dealt with 15mM NH4Cl for 6 (h) Prepared cell lysates were immunoprecipitated by anti-HA antibodies and immunoblotted by anti-G antibodies. All the cell lysates were examined by Western blot for protein expressions (bottom panels).

To explore which SVCV proteins affected by HSC70, pcDNA4-HSC70-Flag plasmid or vector plasmid and plasmid expressing individual viral protein (N, P, M or G) were cotransfected into FHM cells. As shown in **Figure 5A**, HSC70 only reduced expression of SVCV G protein, but did not affect other SVCV proteins. To confirm the suppressive activity of HSC70 on SVCV G protein expression, FHM cells were co-transfected with pcDNA4-HSC70-Flag or vector and pcDNA4-G plasmids. SVCV G protein abundance decreased in a dose-dependent manner after overexpression of HSC70 (**Figure 5B**). Moreover, our findings showed that compared with RNAi control, down-regulation of endogenous HSC70 by RNAi significantly increased the level of SVCV G protein in pcDNA4-G-transfected cells (**Figure 5C**). Consistently, the knockdown of HSC70 also significantly increased SVCV G protein levels in SVCV-infected cells (**Figure 5D**). These data suggested that HSC70 specifically downregulated SVCV G protein expression.

To investigate the role of proteasome-, lysosome-, or autophagosome-dependent pathways in the reduction of SVCV G protein induced by HSC70, the inhibitory effects were evaluated with proteasome inhibitor MG132, lysosome inhibitor NH_4_Cl, and autophagosome inhibitor 3-MA. FHM cells, co-transfected with pcDNA4-HSC70-Flag and pcDNA4-G plasmids, were cultured with or without each inhibitor. From **Figure 5E**, incubation with NH4Cl could restore the level of SVCV G protein in overexpressed HSC70 cells, while MG132 with 3-MA showed no effect. These data demonstrate that HSC70 is involved in SVCV G protein degradation *via* the lysosomal degradation pathway. As shown in **Figure 5F**, yellow signals indicating the overlap of green and red were found in cells co-transfected with the marker proteins of lysosome, SVCV G protein and LAMP2. These findings indicate SVCV G protein introduced into lysosomes in host cells. Furthermore, the role of HSC70 in SVCV G protein ubiquitination in the context of SVCV infection was validated and the SVCV G protein ubiquitination was increased in FHM cells transfected with pcDNA4-HSC70-Flag (**Figures 5G, H**). These results clearly demonstrated that HSC70 facilitates the ubiquitylation of SVCV G protein, leading to SVCV G protein degradation *via* the lysosome.

### MARCH8, HSC70 and SVCV G Proteins Form a Complex

**Figure 6:**
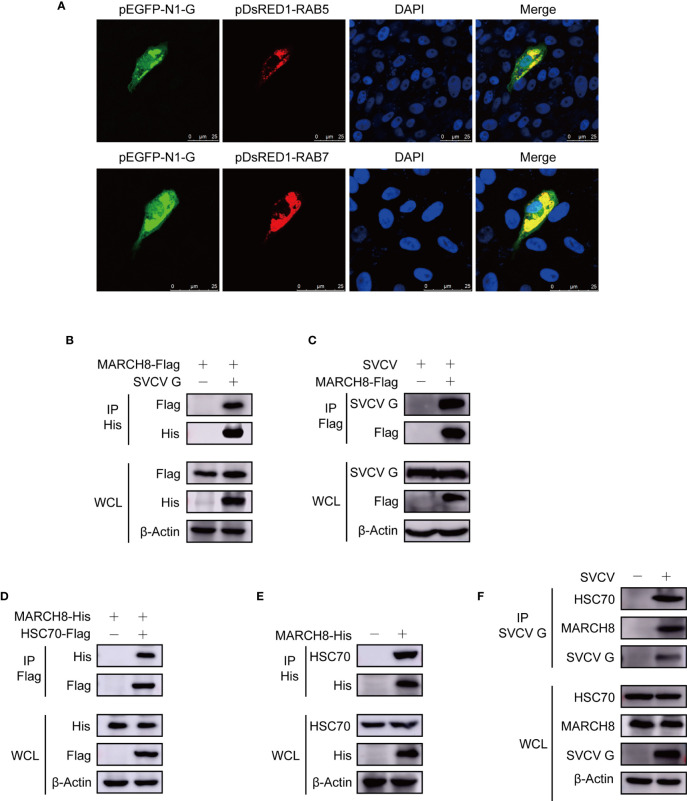
Complex of MARCH8, HSC70 and SVCV G proteins. **(A)** Localization of SVCV G protein to early and late endosomes. FHM cells were transiently co-transfected with early endosomal protein marker pEGFP-N1-G and late endosomal protein marker RAB5-RFP, respectively, and sowed on observation dishes for confocal microscopy. After 24 h, the cells were stained with DAPI after fixing with 4% (v/v) paraformaldehyde. All samples were then examined under a confocal microscope. Green represents SVCV G protein overexpression and red stands for RAB5 or RAB7 overexpressions. Blue staining indicates nucleus. Yellow indicates the co-localization between SVCV G protein and organelle in the merged images. **(B)** Interaction between SVCV G and exogenous MARCH8 proteins. FHM cells were transfected with expressed plasmids. After transfection for 24 h, prepared cell lysates were immunoprecipitated by anti-His antibodies and immunoblotted with anti-His or anti-FLAG antibodies. **(C)** Interaction between SVCV G and exogenous MARCH8 proteins in SVCV-infected cells. FHM cells were transfected with expressed plasmids. After transfection for 6 h, cells were infected by SVCV, with an MOI of 0.05. After 36 hpi, prepared cell lysates were immunoprecipitated by anti-FLAG antibodies and immunoblotted by anti-G or anti-FLAG antibodies. **(D)** Interaction between exogenous HSC70 and MARCH8 proteins. FHM cells were transfected with expressed plasmids. After transfection for 24 h, prepared cell lysates were immunoprecipitated by anti-Flag antibodies and immunoblotted by anti-Flag or anti-His antibodies. **(E)** Interaction between exogenous MARCH8 and endogenous HSC70 proteins. FHM cells were transfected with expressed plasmids. After transfection for 24 h, prepared cell lysates were immunoprecipitated by anti-His antibodies, and immunoblotted with anti-His or anti-HSC70 antibodies. **(F)** Interaction between SVCV G protein with both endogenous MARCH8 and HSC70 proteins to form a complex. FHM cells were infected by SVCV, with an MOI of 0.05. At 24 hpi, prepared cell lysates were immunoprecipitated by anti-G antibodies and immunoblotted with anti-G, anti-MARCH8, or anti-HSC70 antibodies.

To investigate the degradation mechanism of SVCV G protein, subcellular localization of SVCV G protein was examined by confocal microscopy. We co-transfected FHM cells with construct expression G protein fused with EGFP and respective organelle protein markers. As shown in **Figure 6A**, yellow signals indicating the overlap of green and red were found in cells co-transfected with the marker proteins of early endosome and late endosome, SVCV G protein and RAB5 and RAB7, respectively. These findings suggest that SVCV G protein localizes to both early and late endosomes, suggesting that in the host cell, the SVCV G protein was transported into early endosome and late endosome. Given that the E3 ubiquitin ligase MARCH8, which has been identified as a candidate interactor with G protein in our previous study (20), was reported to localize to early endosome and late endosome. Thus, whether MARCH8 interacted with SVCV G protein and acted as an E3 ubiquitin ligase for lysosomal degradation of SVCV G protein was examined. FHM cells were transfected with pcDNA4-G and/or pcDNA4-MARCH8-Flag through immunoprecipitation assays. Therefore, when the lysates of FHM cells expressing pcDNA4-MARCH8-Flag and pcDNA4-G were immunoprecipitated with His antibody, pcDNA4-MARCH8-Flag was easily detected in the precipitate, suggesting that SVCV G protein can interact with exogenous MARCH8 of FHM cells (**Figure 6B**). To further confirm the interaction between MARCH8 and SVCV G protein, we transfected FHM cells with pcDNA4-MARCH8-Flag followed by infection with SVCV, and a Co-IP assay was performed with anti-FLAG antibodies. Our results showed that SVCV G protein was easily detected in the precipitate, confirming the interaction between MARCH8 and SVCV G protein (**Figure 6C**).

Since our report showed that HSC70 interacted with SVCV G protein, which was critical in the degradation of SVCV G protein, it would be interesting to determine whether HSC70 is also involved in MARCH8 in cells. FHM cells were transfected with pcDNA4-MARCH8 and/or pcDNA4-HSC70-Flag through immunoprecipitation assays. When lysates of cells expressing both pcDNA4 -MARCH8 and pcDNA4-HSC70-Flag were immunoprecipitated with FLAG antibodies, pcDNA4-MARCH8 was easily detected in the precipitate, suggesting that MARCH8 can interact with HSC70 of FHM cells (**Figure 6D**). To confirm MARCH8 binding to HSC70, pcDNA4-MARCH8 was expressed in FHM cells to examine its interaction with endogenous HSC70 through a pulldown assay. Binding pcDNA4-MARCH8 to endogenous HSC70 was easily detected in MARCH8-expressing cells (**Figure 6E**). To confirm the interaction between HSC70 and MARCH8 in infected SVCV cells with physiological conditions, FHM cells were infected with SVCV and then subjected to pulldown assay. When lysates of infected SVCV cells were immunoprecipitated with antibodies of SVCV G protein, endogenous HSC70, as well as MARCH8, were detected in the precipitate (**Figure 6F**), indicating that HSC70, MARCH8, and SVCV G protein formed a complex in host cells infected with SVCV.

### MARCH8 Induces SVCV G Protein Degradation *via* Lysosomal Pathway

**Figure 7:**
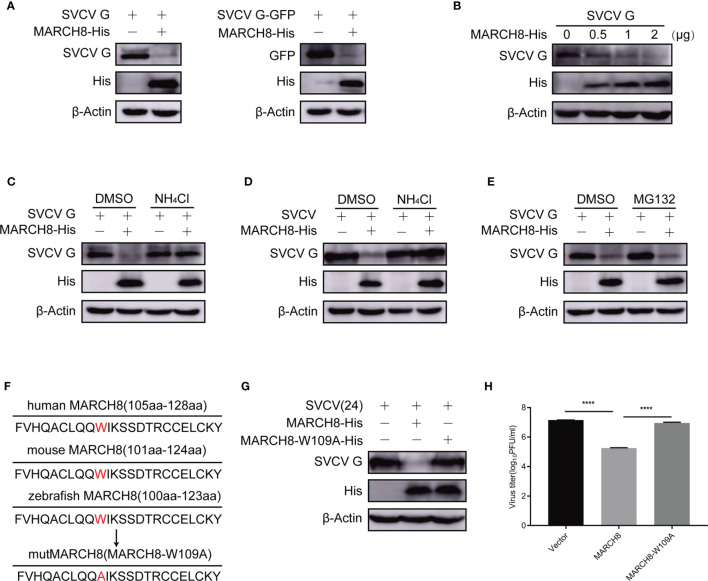
MARCH8-induced degradation of SVCV G protein *via* lysosomal pathway. **(A)** Degrade of SVCV G protein *via* MARCH8. FHM cells were transfected by the indicated plasmids. After transfection for 24 h, protein levels were examined by Western blot. **(B)** MARCH8-induced reduction of SVCV G protein in a dose-dependent manner. The number of pcDNA4-MARCH8 plasmid increased after transfection of pcDNA4-G plasmid into FHM cells. After transfection for 24 h, protein levels were examined by Western blot. **(C)** FHM cells were transfected by the indicated plasmids. After transfection for 18 h, cells were dealt with 15 mM NH_4_Cl or DMSO for 6 h, and protein levels were examined by Western blot. **(D)** FHM cells were transfected by the indicated plasmids. After transfection for 24 h, cells were infected by SVCV, with an MOI of 0.05. At 18 hpi, cells were treated with 15 mM NH_4_Cl or DMSO for 6 h, and protein levels were examined by Western blot with the corresponding antibodies. **(E)** MG132 not affecting MARCH8-induced degradation of SVCV G protein. FHM cells were transfected by the indicated plasmids. After transfection for 18 h, cells were treated with 10μM MG132 or DMSO for 6 h, and protein levels were examined by Western blot. **(F)** Sequence comparison between zebrafish MARCH8 with other indicated species. The mutMARCH8 was generated by inducing a mutation W109A in the wtMARCH8 plasmid with overlap PCR. **(G)** Transfection of cells with MARCH8-W109A abolishes MARCH8-induced degradation of SVCV G protein. FHM cells were transfected with expressed plasmids. After transfection for 24 h, cells were infected by SVCV, with an MOI of 0.05. At 24 hpi, protein levels were examined by Western blot. **(H)** Effects of MARCH8-W109A on SVCV replication. FHM cells were transfected with expressed plasmids. After transfection for 24 h, cells were infected by SVCV, with an MOI of 0.05. At 24 hpi, the supernatant was collected and subjected to plaque assays. ****p < 0.0001.

Since SVCV G protein is characterized by ubiquitination modification and rapid degradation, we examined the effect of MARCH8 on SVCV G protein degradation in FHM cells cotransfected with pcDNA4-G or pEGFP-N1-G and pcDNA4-MARCH8. The protein expression level of SVCV G protein was decreased in FHM cells transfected with pcDNA4-MARCH8 (**Figure 7A**). Cotransfection of FHM cells with pcDNA4-G and increasing amounts of pcDNA4-MARCH8 showed that MARCH8 reduced protein expression of SVCV G protein in a dose-dependent manner (**Figure 7B**). In addition, Western blot analysis of transfected FHM cells with pcDNA4-G with or without pcDNA4-MARCH8 with or without NH_4_Cl, a lysosomal degradation inhibitor showed that the overexpression of MARCH8 significantly reduced SVCV G protein levels. Furthermore, the reduction of the SVCV G protein level mediated by MARCH8 was markedly inhibited by the lysosome inhibitor NH_4_Cl (**Figure 7C**). Similar results were also obtained from an experimental test with SVCV infected FHM cells (**Figure 7D**). Compared with NH_4_Cl, MG132 is a proteasomal inhibitor commonly used, without any inhibitory effect on the degradation of SVCV G protein induced by MARCH8 in pcDNA4-G-transfected cells (**Figure 7E**). These results indicate that MARCH8 contributes to SVCV G protein degradation *via* lysosomal rather than proteasomal pathway.

To explore the role of tryptophan residues of amino acid 109 of MARCH8 in the degradation of SVCV G protein, a tryptophan residue conserved in the RING domain of E3 ubiquitin ligases is known to be essential for binding E2 enzyme and its activity (24). A MARCH8 mutant was constructed with the substitution of tryptophan to alanine at the 109 codon of its N-terminal RING domain (MARCH8-W109A) and the effect of MARCH8 mutant on the degradation of SVCV G protein was examined. Therefore, the mutant MARCH8-W109A reversed the decrease in SVCV G protein levels caused by MARCH8 (**Figures 7F, G** **)**, MARCH8-W109A failed to display an anti-SVCV action (**Figure 7H**), indicating that tryptophan 109 of MARCH8 is vital for its catalytic activity in the degradation of SVCV G protein.

### MARCH8 Facilitates Ubiquitylation of SVCV G Protein

**Figure 8:**
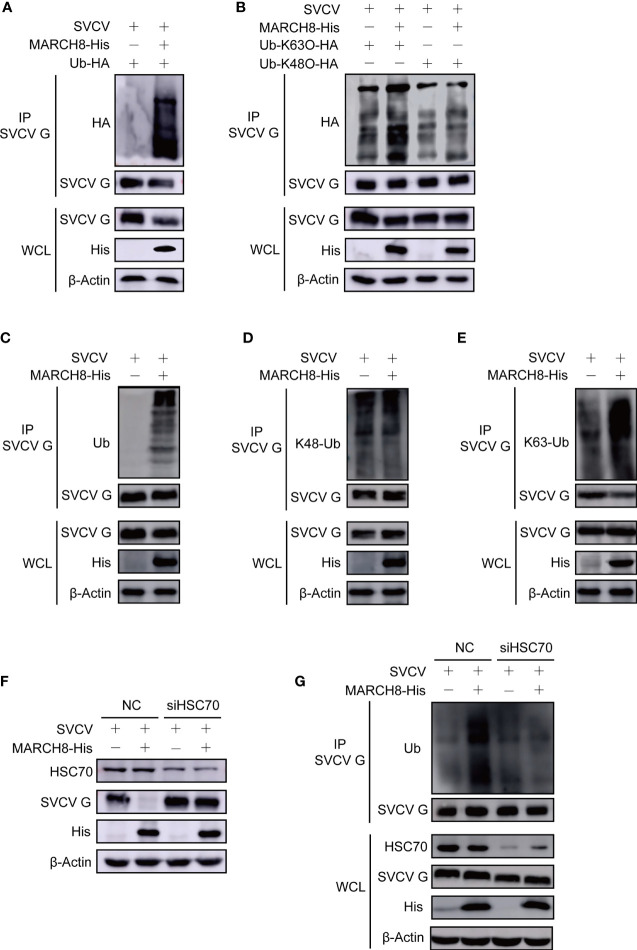
MARCH8 facilitates ubiquitylation of SVCV G protein. **(A)** Enhancement of MARCH8 overexpression to the ubiquitylation of SVCV G protein. Plasmids encoding HA-ubiquitin and pcDNA4-MARCH8 or pcDNA4 were adopted to transfect FHM cells as controls. After transfection for 6 h, cells were infected by SVCV, with an MOI of 0.05. At 18 hpi, cells were treated by 15mM NH_4_Cl for 6 (h) Prepared cell lysates were immunoprecipitated with anti-G antibodies and immunoblotted with anti-G or anti-HA antibodies. All the cell lysates were examined by Western blot for protein expression (bottom panels) **(B)** Enhancement of MARCH8 overexpression to K63-ubiquitylation of SVCV G protein. Plasmids encoding HA-ubiquitin-K63O or HA-ubiquitin-K48O and pcDNA4-MARCH8 or pcDNA4 were adopted to transfect FHM cells as controls. After transfection for 6 h, cells were infected by SVCV, with an MOI of 0.05. At 18 hpi, cells were treated by 15mM NH4Cl for 6 (h) Prepared cell lysates were immunoprecipitated by anti-G antibodies and immunoblotted by anti-G or anti-HA antibodies. All the cell lysates were examined by Western blot for protein expression. **(C–E)** Enhancement of MARCH8 overexpression to the endogenous ubiquitylation of SVCV G protein. Plasmids encoding pcDNA4-MARCH8 or pcDNA4 were adopted to transfect FHM cells as controls. After transfection for 6 h, cells were infected by SVCV, with an MOI of 0.05. At 18 hpi, cells were treated by 15mM NH_4_Cl for 6 (h) Prepared cell lysates were immunoprecipitated with anti-G antibodies and immunoblotted with anti-G or anti-polyubiquitin antibodies, anti-K48-polyubiquitin antibodies and anti-K63-polyubiquitin antibodies. All the cell lysates were examined by Western blot for protein expression (bottom panels)**. (F)** Knockdown of HSC70 mitigating MARCH8-induced degradation of SVCV G protein. Cells were treated by HSC70 or control RNAis with pcDNA4-MARCH8 or pcDNA4. After transfection for 6 h, cells were infected by SVCV, with an MOI of 0.05. At 24 hpi, prepared cell lysates were immunoblotted by anti-HSC70, anti-His or anti-G antibodies. **(G)** Knockdown of HSC70 mitigating MARCH8-induced ubiquitylation of SVCV G protein. Cells were treated by HSC70 or control RNAis with pcDNA4-MARCH8 or pcDNA4. After transfection for 6 h, cells were infected by SVCV, with an MOI of 0.05. At 18 hpi, cells were treated by 15mM NH_4_Cl for 6 (h) Prepared cell lysates were immunoprecipitated with anti-G antibodies and immunoblotted with anti-G or anti-polyubiquitin antibodies. All the cell lysates were examined by Western blot for protein expression (bottom panels).

To explore the role of MARCH8 in the ubiquitylation of SVCV G protein through lysosomal degradation pathway, plasmids encoding hemagglutinin (HA)-ubiquitin, pcDNA4-G, and pcDNA4-MARCH8 or the pcDNA4 control were transfected into FHM cells followed through immunoprecipitation assays. Thus, compared with the control group, MARCH8 overexpression significantly enhanced the ubiquitylation of SVCV G protein in FHM cells (**Figure 8A**). To clarify the mechanism in the degradation of SVCV G protein and the effect of MARCH8 on K63-and K48-linked ubiquitination of SVCV G protein, ubiquitination assays in FHM cells revealed that MARCH8 overexpression can enhance K63-linked ubiquitination for SVCV G protein (**Figure 8B**). Furthermore, we used endogenous Ub antibody to detect MARCH8-mediated ubiquitination of SVCV G protein. The result showed that MARCH8 overexpression significantly enhanced the total ubiquitylation and K63-linked ubiquitination of SVCV G protein in FHM cells (**Figures 8C–E**), which clearly show that MARCH8 can mediate SVCV G protein ubiquitylation, resulting in the degradation of SVCV G protein *via* the lysosome.

To validate whether HSC70 induces SVCV G protein degradation by interacting with MARCH8, the effect of MARCH8 on the degradation of SVCV G protein was examined in SVCV-infected cells with the knockdown of HSC70. As shown in **Figure 8F**, the knockdown of HSC70 by RNAi significantly inhibited the reduction of SVCV G protein levels mediated by MARCH8 compared to that of RNAi controls, indicating that SVCV G protein degradation induced by MARCH8 is associated with HSC70. The effect of MARCH8 on the ubiquitination of SVCV G protein was examined in HSC70 knockdown and SVCV-infected cells. As shown in **Figure 8G**, the knockdown of HSC70 by RNAi significantly inhibited the ubiquitination of SVCV G protein mediated by MARCH8 compared to negative control. These results reveal that as a scaffold protein, HSC70 enables MARCH8 to target SVCV G protein for ubiquitination and degradation *via* the lysosomal pathway.

### MARCH8 Inhibits SVCV Infection

**Figure 9:**
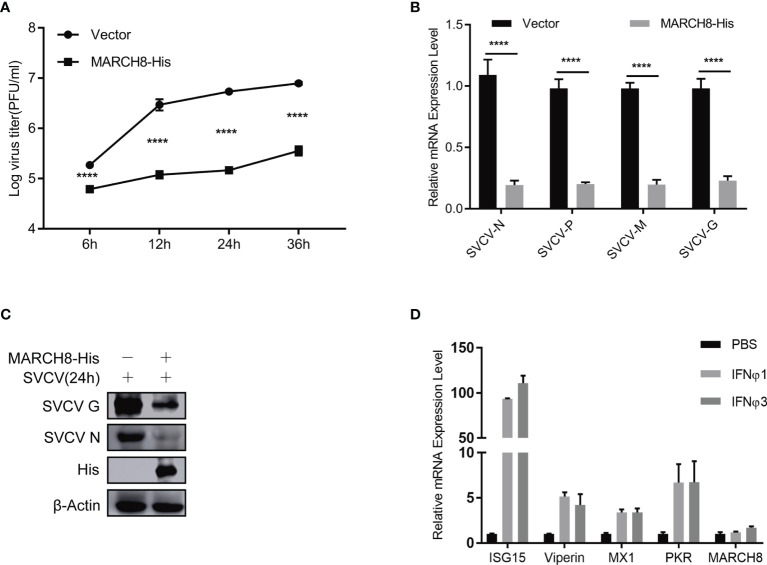
MARCH8 inhibits SVCV infection. **(A–C)** Overexpression of MARCH8 suppresses SVCV growth in FHM cells. FHM cells were transfected with MARCH8 plasmid and empty vector (2μg each) for 24 h, followed by infection with SVCV, with an MOI of 0.05. Cell samples and the supernatant collected at the indicated time points were analyzed by qRT-PCR **(A)**, Western blot **(B)**, or plaque formation assay **(C)**. **(D)** MARCH8 is not an IFN-stimulated gene. RT-qPCR analysis of MARCH8 and ISGs (ISG15, Viperin, MX1 and PKR) mRNA levels in FHM cells treated with 100 ng/ml of IFNϕ1, 100 ng/ml of IFNϕ3. ****p < 0.0001.

To verify the potential inhibition of MARCH8 on SVCV replication, FHM cells were transfected with increasing amounts of pcDNA4-MARCH8 plasmids, followed by infection with equal amounts of SVCV (MOI of 0.05). At 24 hpi, the levels of viral RNA and the abundance of viral proteins were examined (**Figures 9A, B** **)**. At 6, 12, 24 or 36 hpi, viral titers were determined (**Figure 9C**). RT-PCR, Western blot and viral titer analysis indicate significantly suppressed SVCV replication by MARCH8 overexpression. To investigate whether MARCH8 is an interferon stimulated gene (ISG), we treated FHM cells with type I (IFNϕ1 and IFNϕ3). In contrast to well-known ISGs (ISG15, Viperin, PKR and MX1), in FHM cells, type I interferon only slightly up-regulated MARCH8 expression **(** **Figure 9D** **)**, indicating that fish MARCH8 isn’t a new ISG.

### Lysine Residues in CT Domain of SVCV G Protein Are Involved in MARCH8-Induced Degradation

**Figure 10:**
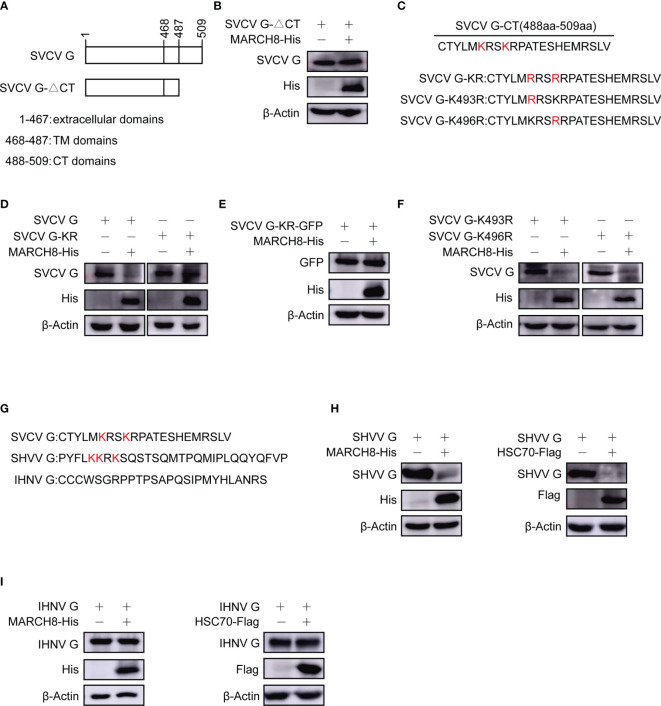
Reduction of lysines in SVCV G protein with CT domain involved in MARCH8-induced degradation. **(A)** Schematic presentation for functional domains of SVCV G protein and the mutants used in the study. **(B)** FHM cells were cotransfected with plasmids expressing His-tagged MARCH8 and SVCV G-△CT. After transfection for 24 h, protein levels were examined by Western blot. **(C)** Schematic presentation of CT domains of SVCV G protein and the Lysine mutants used in the study. **(D–F)** FHM cells were cotransfected with plasmids expressing His-tagged MARCH8 and SVCV G, SVCV G-KR **(D)**, SVCV G-KR-GFP **(E)**, SVCV G-K493R or SVCV G-K496R **(F)**. After transfection for 24 h, protein levels were examined by Western blot. **(G)** Schematic presentation of CT domains of 3 viruses belonging to the Rhabdoviridae family (SVCV, SHVV, and IHNV) G protein used in the study. **(H, I)** FHM cells were cotransfected with plasmids expressing His-tagged MARCH8 or Flag-tagged HSC70 and SHVV G **(H)** or IHNV G **(I)**. After transfection for 24 h, protein levels were examined by Western blot.

To identify the key domains within SVCV G protein required for its degradation, different plasmids expressing SVCV G protein mutants, CT domain-lacking SVCV G protein (SVCV G-ΔCT) were constructed (**Figure 10A**). The plasmids expressing His-tagged MARCH8 and plasmids expressing mutant SVCV G protein were cotransfected into FHM cells. Western blot assay showed that the deletion of the CT domain completely abolished the degradation of G protein (**Figure 10B**), suggesting that the CT domain is required for the degradation of SVCV G protein. We constructed plasmids expressing SVCV G protein mutants, lysine residues completely mutation in CT domain of SVCV G protein (SVCV G-KR) and lysine residues single mutation in CT domain of SVCV G protein (SVCV G-K493R and SVCV G-K496R) (**Figure 10C**). To explore the resistance of KR mutant of SVCV G protein to the degradation induced by MARCH8, FHM cells were co-transfected with MARCH8 plasmid expressing His and with mutant SVCVG protein, and a Western blot assay was performed. As shown in **Figures 10D, E**, the lysine residues mutation of the CT domain completely abolished the degradation, suggesting that the lysine residues in CT domain are required for the degradation of SVCV G protein. As shown in **Figure 10F**, the lysine residues single mutation of the CT domain did not abolish the degradation, this means that as long as there is a lysine residue in the CT domain of SVCV G protein, the degradation will occur. We also compared the CT region of the G protein of the other two *Rhabdoviruses* (snakehead vesiculovirus and infectious hematopoietic necrosis virus), and similar lysine residues was found in the CT region of SHVV but not IHNV (**Figure 10G**). To determine whether SHVV G protein and IHNV G protein will be degraded by MARCH8, the plasmids expressing His-tagged MARCH8 and plasmids expressing G protein were cotransfected into FHM cells, and a Western blot assay was performed. As shown in **Figures 10H, I**, SHVV G protein was degraded by MARCH8 and HSC70 but IHNV G protein was not degraded. These results indicate that lysine residues can be specifically ubiquitinated by MARCH8 in CT domain of SVCV G protein, leading to lysosomal degradation.

## Discussion

SVCV, a single-stranded RNA virus belonging to Rhabdoviruses, is an important pathogens in wild and cultured fish all over the North America, Asia, and Europe (25, 26). SVCV infection can induce the apoptosis in cultured cells and fish infected tissues. It has been previously reported SVCV G protein involved in syncytium formation and the apoptosis in affected cells, representing one of the target for host antiviral response. In the study, the degradation of SVCV G protein was found to be related to HSC70 for the first time. Also, HSC70, as a scaffold for interactions with MARCH8 and SVCV G protein, was found to facilitate MARCH8-mediated SVCV G protein degradation.

As a multifunctional protein (27), the role of HSC70 in many viral infections was reported previously (21, 22). HSC70 was identified as a potential receptor or co-receptor for NNV (28). Additionally, studies showed that it was involved in the entry steps of virus, such as rotavirus, dengue virus, Japanese encephalitis virus, polyomavirus, and hepatitis C virus (HCV) (29–34). Although it has been widely believed that in host cells, viruses hijack chaperone pathways facilitate their propagation (35–37), interestingly, cell chaperones have been in line with antiviral activity sometimes. Previous report indicates that HSP40 proteins critical in the instability of the HCV core and X proteins (38). DNAJA3 has an important antiviral effect on FMDV by degrading VP1 and restoring IFN-β signaling pathway (39). However, HSC70’s effect on the infection of SVCV remains unclear. The negative effects of HSC70 in SVCV replication are reported here for the first time. We indicate that HSC70 serves as a scaffold for both MARCH8 and SVCV G protein that allows the E3 ligase targeting SVCV G protein for ubiquitylation and degradation to suppress SVCV infection, which suggests a new function of HSC70.

This study also examined E3 ubiquitin ligase responsible for ubiquitin-dependent degradation of SVCV G protein and showed that MARCH8 of E3 ubiquitin ligase had interactions with SVCV G protein. MARCH8 overexpression markedly facilitated the ubiquitylation and degradation of SVCV G protein in host cells. These new findings have clearly established the role of MARCH8 as an E3 ligase for SVCV G protein ubiquitylation, and the E3 is the only ligase identified so far for the lysosomal degradation of SVCV G protein. Moreover, we found that HSC70 interacted with both SVCV G protein and MARCH8 and that HSC70 knockdown mitigated SVCV G protein levels reduced by MARCH8. These findings indicate that HSC70 can induce SVCV G protein degradation *via* MARCH8, the E3 ligase for SVCV G protein ubiquitylation.

As we know, MARCH8 has different effects in cells of inflammation, innate immunity and cancers (16, 24, 40). In this study, the possible function of MARCH8 in the degradation of SVCV G protein was explored. Our data show that MARCH8 promotes SVCV G protein’s ubiquitin-lysosomal degradation by interacting with HSC70 and SVCV G proteins, with an important role in host defense against the infection of SVCV. Besides SVCV G protein, the degradation and maturation of several viral proteins, including VSV G protein, EBOV GP protein, IAV HA protein, HIV-1 Env protein and so on can also be mediated by MARCH8 (19, 41). For the importance of viral proteins’ expressions for the pathogenesis of these pathogens, March8 may undergo lysosomal degradation through ubiquitination of viral proteins, with an important role in host defense against viral infection. In this paper, it was found that MARCH8 can not only ubiquitinate SVCV G protein, but also promote its lysosomal degradation, its activity is dependent on the RING domain, because W109A mutant with a dead RING domain is inactive. These findings indicate that the activity of MARCH8 is dependent on ubiquitination pathways, and lysine residues in SVCV G protein with CT domain participate in the MARCH8-induced degradation. Thus, MARCH8-induced degradation of SVCV G protein is similar to MARCH proteins downregulating immune receptors and viral proteins on the cell surface, where MARCH proteins usually ubiquitinate lysine residues in the cytoplasmic tail of them, converting them from in endosomes from the present to the last and lysosomes for degradation when they are endocytic on the cell surface (42).

It was reported previously that BST-2 regulates the mitochondrial antiviral signaling protein (MAVS) level through interacting with MARCH8. Thus, as a suppressor, MARCH8-BST-2 complex inhibits the continuous activation of RIG-I-like receptor-mediated type I IFN signaling through a negative feedback loop (43). Kong et al. reported the inhibition of MARCH8-BST-2 complex on the replication of PEDV (44). Similar to those reports, in this study, it was also found that SVCV G protein can be degraded by HSC70 *via* MARCH8, indicating that MARCH8 might affect cellular processes by being employed *via* multiple proteins.

In summary, our data provided insight of a novel mechanism of host defense in which, HSC70, as a scaffold protein, interacted with E3 ligase MARCH8 and SVCV G proteins, which initiates MARCH8-mediated lysosomal degradation of G protein and thus suppresses SVCV replication. The above results reveal that HSC70-MARCH8 complex has a novel role in the antiviral response of host, providing insights for developing therapeutics targeting SVCV.

## Data Availability Statement

The datasets presented in this study can be found in online repositories. The names of the repository/repositories and accession number(s) can be found in the article/**Supplementary Material**.

## Author Contributions

XL: Conceptualization and methodology. CL: Validation, investigation, and writing - original draft. LS: Formal analysis and data curation. YG: Resources. YL: Writing - review & editing. JY: Validation and supervision. All authors contributed to the article and approved the submitted version.

## Funding

This work was supported by the Natural Science Foundation of China (32173018, 31972834), the National Key Research and Development Program of China (2018YFD0900505).

## Conflict of Interest

The authors declare that the research was conducted in the absence of any commercial or financial relationships that could be construed as a potential conflict of interest.

## Publisher’s Note

All claims expressed in this article are solely those of the authors and do not necessarily represent those of their affiliated organizations, or those of the publisher, the editors and the reviewers. Any product that may be evaluated in this article, or claim that may be made by its manufacturer, is not guaranteed or endorsed by the publisher.
