# Supplementary material for: HSC70 Inhibits Spring Viremia of Carp Virus Replication by Inducing MARCH8-Mediated Lysosomal Degradation of G Protein
Source: Front Immunol. 2021 Sep 29;12:724403. doi: 10.3389/fimmu.2021.724403 (PMC8511485; doi:10.3389/fimmu.2021.724403)
Supplement: Supplementary file 1 [file Table_1.doc]

**Table 1.**

Primers of this study.

| Application | Prime Name | Sequence (5′–3′) |
| --- | --- | --- |
| qRT-PCR | qTBP-F | TTACCCACCAGCAGTTTAG |
| qRT-PCR  qRT-PCR  qRT-PCR  qRT-PCR  qRT-PCR | qTBP-R  qCc40S-F  qCc40S-R  qMARCH8-F  qMARCH8-R | ACCTTGGCACCTGTGAGTA  CCGTGGGTGACATCGTTACA  TCAGGACATTGAACCTCACTGTCT  CTCGGTCACTTTCCACG  CTTGAGTCTCCTCCACAACT |
| qRT-PCR | SVCV-G-F | CGACCTGGATTAGACTTG |
| qRT-PCR | SVCV-G-R | AATGTTCCGTTTCTCACT |
| qRT-PCR | SVCV-M-F | TACTCCTCCCACTTACGA |
| qRT-PCR  qRT-PCR  qRT-PCR  qRT-PCR  qRT-PCR  qRT-PCR  qRT-PCR  qRT-PCR  qRT-PCR  Plasmid construction  Plasmid construction  Plasmid construction  Plasmid construction  Plasmid construction  Plasmid construction  Plasmid construction  Plasmid construction  Plasmid construction  Plasmid construction  Plasmid construction  Plasmid construction  Plasmid mutation  Plasmid mutation  Plasmid mutation | SVCV-M-R  SVCV-N-F  SVCV-N-R  qISG15-F  qISG15-R  qPKR-F  qPKR-R  qViperin-F  qViperin -R  HSC70-Flag-F  HSC70-Flag-R  HSC70-His-F  HSC70-His-R  MARCH8-His-F  MARCH8-His-R  MARCH8-Flag -F  MARCH8-Flag-R  SVCV G-F  SVCV G-R  SVCV G-EGFP-F  SVCV G-EGFP-R  SVCV G-△CT-F  SVCV G-△CT-R  SVCV G-KR-F | CAAGAGTCCGAGAAGGTC  GCGGTTTTCTGTATGTGTCTC  CTCTGCCAAATCACCATACTC  TAATGCCACAGTCGGTGAA  AGGTCCAGTGTTAGTGATGAGC  ACCTGAAGCCTCCAAACATA  GCATTCGCTCATCATTGTC  GCAAAGCGAGGGTTACGAC  CTGCCATTACTAACGATGCTGAC  CCCAAGCTTGCCACCATGTCCAAGGGACCAGCTGTTGGTATT  CCGCTCGAGTTACTTATCGTCGTCATCCTTGTAATC  GTCGACCTCCTCGATGGTTGGG  CCCAAGCTTGCCACCATGTCCAAGGGACCAGCTGTTGGTATT  CCGCTCGAGGTCGACCTCCTCGATGGTTGGG  CGGAATTCATGAACATGCCACTGCACCAGATCT  CCGCTCGAGCACGTGAAGGATCTCCATACTG  CGGAATTCATGAACATGCCACTGCACCAGATCT  CCGCTCGAGTCACTTATCGTCGTCATCCTTGTAATC CACGTGAAGGATCTCCATACTG  CCCAAGCTTGCCACCATGTCTATCATCAGCTACATCGCAT  CCGCTCGAGAACTAAAGACCGCATTTCGTGT  CCGCTCGAGGCCACCATGTCTATCATCAGCTACATCGCAT  CCCAAGCTTAACTAAAGACCGCATTTCGTGT  CCCAAGCTTGCCACCATGTCTATCATCAGCTACATCGCAT  CCGCTCGAGAGCAACACAGCATCTGATGAGAAGA  CCCAAGCTTGCCACCATGTCTATCATCAGCTACATCGCAT |
| Plasmid mutation  Plasmid mutation  Plasmid mutation  Plasmid mutation  Plasmid mutation  Plasmid construction  Plasmid construction  Plasmid construction  Plasmid construction | SVCV G-KR-R  SVCV G-K493R-F  SVCV G-K493R-R  SVCV G-K496R-F  SVCV G-K496R-R  SHVV-G-F  SHVV-G-R  IHNV-G-F  IHNV-G-R | CCGCTCGAGAACTAAAGACCGCATTTCGTGTGATTCTGTTGCAGGCCGTCTACTCCTCCTCATCAAATA  CCCAAGCTTGCCACCATGTCTATCATCAGCTACATCGCAT  CCGCTCGAGAACTAAAGACCGCATTTCGTGTGATTCTGTTGCAGGC  CGTTTACTCCTCCTCATCAAATA  CCCAAGCTTGCCACCATGTCTATCATCAGCTACATCGCAT  CCGCTCGAGAACTAAAGACCGCATTTCGTGTGATTCTGTTGCAGGCCGTCTACTCCT  CGGAATTCGCCACCATGAAATCAATCATTGCACTTACGT  GCTCTAGAGGGAACAAATTGATACTGCTGCAAA  CCCAAGCTTGCCACCATGTACACCATGATCACCACTCCGC  CCGCTCGAGGGACCGGTTTGCCAGGTGATACATG |
